# The moderating effect of self-efficacy on supervisory support and organizational citizenship behavior

**DOI:** 10.3389/fpsyg.2022.961270

**Published:** 2022-09-20

**Authors:** Meor Rashydan Abdullah, Walton Wider

**Affiliations:** Faculty of Business and Communications, INTI International University, Nilai, Malaysia

**Keywords:** supervisory support, organizational citizenship behavior, self-efficacy, public sector, SEM-PLS

## Abstract

The study aims to examine the moderating effect of self-efficacy on supervisory support and organizational citizenship behavior (OCB). An individual’s self-efficacy is defined as their belief in their own ability to successfully complete a goal or task, which influences their motivation, persistence, and decision-making. This study is based on the Conservation of Resource Theory, which holds that personal resources such as self-efficacy can influence employees’ perceived support and extra-role behavior (OCB). The data were collected from 618 employees in four public sector organizations in Putrajaya, Malaysia through a questionnaire survey and analyzed using the partial least squares structural equation modeling (PLS-SEM) technique. Resultantly, supervisory support demonstrated a significant positive relationship with OCB. The results suggested that personal resources, such as self-efficacy increase the level of OCB with lower or higher perceived supervisory support. The results highlighted that self-efficacy strengthens supervisory support relations with OCB when supported by employees’ self-belief and confidence. It is critical to investigate the role of self-efficacy because industries must constantly change, and employees must have self-efficacy resources to continuously improve and sustain their performance level. The findings can contribute to the literature and open new avenues for future research.

## Introduction

Over the years, Organizational Citizenship Behavior (OCB) has gained considerable attention, specifically among researchers, management scholars, and practitioners (Kaur et al., 2020; Kaur and Randhawa, 2021). In recent decades, OCB research has become the most widespread topic in the organizational sciences (Klotz et al., 2018). The OCB is an important indicator which could directly contribute to an individual and the overall organizational efficiency and effectiveness (Acaray and Akturan, 2015). Organ (1997) defined OCB as “any discretionary work-related behavior that goes beyond routine duties and supports one’s social or psychological environment.” The five primary dimensions of OCB are (Organ et al., 2006) Altruism: assisting other colleagues (co-worker or supervisor), Civic virtue: an employee’s voluntary participation and involvement in organizational affairs, Conscientiousness: individual doing beyond what is required to meet the minimum requirements, Courtesy: individuals’ good deeds and respect to others, and Sportsmanship: individuals’ focus on positive aspects instead of negative aspects of the organization (Basu et al., 2017). Although OCB is not directly or explicitly part of the formal reward system, the behavior encourages functioning and overall organizational effectiveness.

Empirical research on OCB determinants has been widely identified and covers various aspects related to attitudinal variables, individual characteristics, and the work environment (Easo et al., 2020). Employee development outlines the importance of interactions between organizations, managers, and employees by emphasizing a supportive working environment, such as organizational support and supervisory support (Mylona and Mihail, 2020). Studies on supportive working environment and OCB have examined the effect of organizational support (Reader et al., 2017; Han et al., 2019; Jehanzeb, 2020; Andriyanti and Supartha, 2021), co-worker support (Pasamehmetoglu et al., 2017), and supervisory support (Yadav and Rangnekar, 2015; Tang and Tsaur, 2016; Akram et al., 2018; Shu et al., 2018; Abdullah and Marican, 2020). Most studies on OCB were conducted in developed countries, such as the United States and European countries. Hence, the literature review highlighted several OCB studies in Asian countries, such as China, Taiwan, South Korea, India, Indonesia, and the Philippines (Gan and Yusof, 2020). Most past Malaysian studies were conducted in private sectors (Yin Yin Lau et al., 2020), academic institutions (Ramlee et al., 2016), and public services (Nik Nazli and Sheikh Khairudin, 2018), which involved samples that were not from governmental organizations. Previous researchers also noted a lack of studies in Southeast Asia countries, particularly in Malaysia (Yin Yin Lau et al., 2020).

Supervisory support has been recognized as one of the important antecedences that influence employee OCB (Zhao and Zhou, 2019). Empirical research on supervisory support is vital as it is a primary factor that forms distinguishable employees’ attitudes in the workplace (Kaur and Randhawa, 2021). Supervisory support can be defined as “individuals’ beliefs that supervisor offers them work-related assistance to aid in the performance of their job” (Susskind et al., 2003; Pasamehmetoglu et al., 2017). The concept of perceived supervisory support is to what extent supervisors are considerate and assist employees’ effort in performing a task and appreciate their contribution to the organization (Pasamehmetoglu et al., 2017). Although past studies have confirmed a positive relationship between supervisory support and OCB, a gap exists within the system and no process has accurately identified the direct impact of supervisory support on employees’ OCB (Shim and Faerman, 2015; Yadav and Rangnekar, 2015). Most studies on the effect of supervisory support on behavioral outcomes have been conducted in a Western context, such as psychological capital (Bhanthumnavin, 2003; Paterson et al., 2014; Nisula, 2015; Sihag and Sarikwal, 2015; Ahmed et al., 2017), job satisfaction (Ngah et al., 2010; Adebayo and Ogunsina, 2011; Mehboob et al., 2011; Bagger and Li, 2014; Mazumder et al., 2016), and work engagement (Eisenberger et al., 2016; Ibrahim M. A. et al., 2016; Ibrahim et al., 2019; Ling Suan and Mohd Nasurdin, 2016). Significant studies have supported the predictive ability of supervisory support and OCB (Tang and Tsaur, 2016; Kaur and Randhawa, 2021). Nonetheless, few studies have examined the subject in the non-Western context (Ladebo, 2008; Yadav and Rangnekar, 2015; Tang and Tsaur, 2016; Akram et al., 2018), specifically in developing countries such as Malaysia (Abdullah and Marican, 2020). Podsakoff et al. (2017) proposed that research on supervisory support should be reconsidered under a different context and include the significance of the concept in predictive behavioral workplace outcomes.

Thus, self-efficacy is a personal resource and a belief in one’s ability to perform specific tasks (Cinamon, 2006). According to Choi et al. (2021), self-efficacy might be considered an important individual-level job resource which based on employment characteristics. Self-efficacy, which reflects people’s perceptions of social and organizational situations, can also influence innovative behavior. Positive energy that self-efficacy brings may involve exhibiting pleasant attitude toward coworkers, superiors, subordinates, or the organization in order to enhance pro-social behavior (Ullah et al., 2021). On the other hand, the effects of diverse proactive behavior when self-efficacy resources are present have not yet been investigated (Aftab and Waheed, 2021). The objective of this study is to define how self-efficacy moderates the relationship between supervisory support and OCB. Hence, OCB is positively relation to high job performance, cost reduction, improve operational efficiency, employees retention and customer satisfaction (Podsakoff et al., 2009).

Few studies highlight the role of self-efficacy as a moderator on the effect of organizational support on employees’ behavioral outcomes. The main purpose of this section is to classify the rationale offered by previous research that hypothesized self-efficacy as a moderating variable. Pradhan et al. (2020) noted that limited studies had predicted the relationship between self-efficacy and citizenship behavior, specifically in an organizational setup. Moreover, understanding the conditions of self-efficacy and its influence on job attitudes and behavior is crucial as the absence of contextual factors such as OCB would overestimate the effects of self-efficacy, which creates a misleading assumption on the reality of self-efficacy (Ozyilmaz et al., 2017).

In this study, the Conservation of Resource theory was chosen as the fundamental theoretical model (Hobfoll, 1989; Halbesleben et al., 2014). The theory describes how people strive to acquire, protect, retain, and maintain their resources (Halbesleben et al., 2014). Resources can be defined as “anything perceived by an individual to help them achieve their goals” (Halbesleben et al., 2014). Other variables have been integrated within the COR theory to better explain the role of personal resources on individual behavior based on the current research context and background (Testa et al., 2018; He et al., 2019; Marić et al., 2019; Sri Ramalu and Janadari, 2020). Current research demonstrating employees’ perceived support and self-efficacy as resources was incorporated into the COR theory to create a comprehensive theoretical model to understand individuals’ motivation to participate in extra-role activities such as OCB (de Andrade et al., 2017; Montani and Dagenais-Desmarais, 2018; Gan and Yusof, 2020; Nguyen et al., 2022).

Despite extensive research on OCB, lack of empirical research with supervisory support has been conducted in Malaysia’s public sector. The literature supports that there is still a need for a study investigating the relationship between supervisory support and OCB. Specifically, there is limited research in the Malaysian context that examines the moderating role of self-efficacy in the relationship between supervisory support and employee OCB. This study also attempts to bridge the gap between earlier studies and the current argument facing the public sector from the perspective of human resources. However, the current body of research provides a vast scope for examining the impact of supervisory support and OCB (Chen et al., 2008; Ladebo, 2008; Yadav and Rangnekar, 2015; Tang and Tsaur, 2016; Akram et al., 2018; Kaur and Randhawa, 2021).

## Literature review

### Supervisory support

Eisenberger et al. (2002) defined supervisory support as the degree to which employees form a general impression that their supervisors appreciate their contributions, support, and care for their subordinates. A recent definition defined supervisory support as employees’ belief regarding the support and recognition received from supervisors in exchange for the employees’ efforts (Khan et al., 2015). Mazumder et al. (2016) added that supervisory support provides administrative support, including good characteristics, such as attainability, help, support, caring, flexibility, knowledge, experience, and understanding. Supervisory support comprises three forms: emotional support, informative support, and material support (Bhanthumnavin, 2003). Hence, supervisory support is a job resource that is an essential determinant of motivational states, which can increase employees’ good impression of the organization, thus causing employees to reciprocate by performing positive attitudes that encourage greater OCB (Bakker and Demerouti, 2007). Furthermore, supervisors who recognize and contribute to the improvement of the work process create a positive perception of support in the minds of their subordinates and strengthen the high quality of the employee-supervisor relationship (Paillé et al., 2013). The study will then discuss the effect of antecedent on OCB as well as the effect of moderating factors on employees’ supervisory support and OCB relationship.

### Organizational citizenship behavior and antecedents

Organ (1997) described OCB as the “contribution to the maintenance and enhancement of the social and psychological context that support task performance.” Organ later redefined OCB in 2006 as “discretionary individual behavior, not directly or explicitly recognized by the formal reward system and that in aggregate promotes the effective functioning of the organization” (Organ et al., 2006). Recently, the author reconceptualized OCB as a discretionary behavior of cooperation and contributions that participants view as a function of job satisfaction and perceived fairness (Organ, 2018). The antecedents of OCB derived from the study model are based on the conservation of resources (COR) theory (Hobfoll, 1989) which has gained prominence in studies of organizational behavior (Hobfoll et al., 2018).

Hence, supervisory support aligns with the COR theory and is a work-related resource (Bakker et al., 2008) that demonstrates supportive behavior toward subordinates within and outside work life where the subordinates will reciprocate with a high level of identification, compliance, and gratitude by engaging actively in OCB within the organization (Wu et al., 2014). Employees that perceive supervisory support are more likely to invest their available resources, thus engaging in citizenship behavior to gain further resource (Hobfoll et al., 2018). Therefore, the current study examined supervisory support as a predictor of employee OCB in Malaysian public institutions based on the previous COR theory discussion.

Past public sector studies have provided empirical evidence that supervisory support is a crucial predictor of employee OCB (Tang and Tsaur, 2016). For instance, Dai et al. (2018) investigated 612 employees from the hotel industry and discovered that supervisory support is positively related to OCB Pasamehmetoglu et al. (2017) revealed similar results by studying 243 restaurant employees in Turkey. In Malaysia, Ibrahim R. M. et al. (2016) examined 282 public service employees and discovered that supervisory support is positively related to OCB.

Based on the discussion, the study proposed that employees who perceived supervisory support are more likely to feel positive, motivated, and willing to engage in extra-role behavior, such as OCB. Thus, the study presents the following hypothesis:

*Hypothesis 1*: There is a positive relationship between supervisory support and OCB.

### Self-efficacy as a moderator

In line with the COR theory (Hobfoll, 1989; Halbesleben et al., 2014), employees who perceive high supervisory support may behave beyond their task requirements. The study proposed that supervisory support is positively related to demonstrating active OCB, such as performing beyond an assigned task, participating in the organizational activity, and assisting colleagues in solving a work-related issue, which are behaviors that benefit the individual and organization (William and Anderson, 1991). The COR theory describes self-efficacy as a personal resource (Bandura, 1997, 2006). Self-efficacy is defined as “individuals’ judgments of their capabilities to organize and execute course of action required to attain designated types of performances” (Bandura, 1986; Halbesleben et al., 2014). Hence, employees with low self-efficacy are more affected by low supervisory support but are easily influenced by increased supervisory support.

Contrarily, high self-efficacious employees with perceived supervisory support can maintain and perform extra-role tasks and additional work engagement. Employees with low self-efficacy may give up quickly when the situation is against their favor despite supervisory support. Employees who perceive the organizational environment as highly supportive, fair, and honest and receive high supervisory support tend to feel that their efforts are being recognized and are willing to expand their current role to include extra-role behavior outside their job description. High self-efficacious employees expect high self-belief in completing the task at hand (aligned with resource caravans in the COR theory) (Halbesleben et al., 2014) which strengthens the relationship between supervisory support and OCB.

Low self-efficacious employees exhibit a weaker connection between supervisory support and OCB due to anxiety and withdrawal behaviors. Additionally, employees perceive high support as they believe and have confidence in their supervisor to recognize and appreciate their contribution and effort in performing the tasks given (Mulki et al., 2008). Hence, the motivation to engage in OCB will increase among those who perceive high supervisory support under the condition of high self-belief. Moreover, Tsai and Lin (2014) suggested the inclusion of self-efficacy in a research model for OCB antecedents in a non-profit organization, such as the public sector. Therefore, the study proposes the following hypothesis:

*Hypothesis 2*: The positive relationship between supervisory support and OCB is stronger in individuals with higher self-efficacy than lower self-efficacy.

### Research model

**FIGURE 1:**
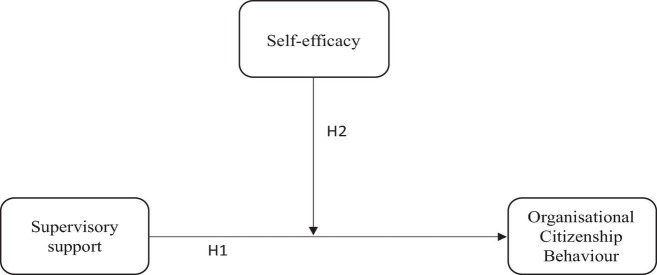
Conceptual framework.

Figure 1 illustrates the proposed study model. The main objective is to test the moderating effect of self-efficacy on the relationship between supervisory support and OCB based on H2. Thus, the direct relationship in H1 was also tested.

## Materials and methods

### Sample and data collection

In federal government organizations in Putrajaya, Malaysia, there were 9,050 Administrative and Diplomatic Officers (ADO) in grades M41-M54 from the Professional and Management levels (Sanali et al., 2013; Abdullah and Kamil, 2020). This study’s unit of analysis is the ADO because this position is responsible for formulating policies and implementing development strategies (Masrek et al., 2013).

A cross-sectional survey of employees at the professional and management levels was conducted. The study used a population sampling strategy, which included the entire population as its sample, to reduce the possibility of a low response in a setting with a small sampling frame (Sekaran and Bougie, 2016). Thus, the specific target population was identified, and information was collected from that population as opposed to the most accessible respondents (Sekaran and Bougie, 2016). In particular, the target respondents are ADOs at the professional and managerial levels within the organization. Any employees who differed from the position scheme and were below it were ineligible to participate in the study.

To determine the sample size for structural equation modeling, the rule of thumb for minimum sample size is 100–150 (Anderson and Gerbing, 1988; Hair et al., 2017). In addition, the current sample size exceeds the minimum of 74 recommended by G*Power analysis, which uses a medium effect size of 0.15. Consequently, a sample size of 618 was deemed adequate for this study because it exceeded the recommended range.

Overall, 1,190 self-administered questionnaires were sent to the Human Resource Department to be distributed to the respondents. Only 714 out of the 1,190 questionnaires were returned after 4-month data collection period from Feb 2018 until June 2018. After removing the questionnaires with over 15% missing value (Hair et al., 2017), 618 questionnaires with a 51.9% response rate were retained to test the study hypotheses. According to Mellahi and Harris (2016), there is no agreed-upon minimum response rate, and there are varying academic perspectives regarding the response rate. For instance, Malhotra and Grover (1998) stated that a response rate of less than 20% was undesirable for research, while Anseel et al. (2010) suggested that an acceptable range for response rate could range from 30 to 70%. Hence, it is critical for achieving the desired response rate, which can provide confidence in the data representativeness and ensure sufficient study validity (Mellahi and Harris, 2016). In this current study, the 618 total responses fulfill the minimum sample size requirement for PLS-SEM analysis as it exceed the range of sample size of 150, as Anderson and Gerbing (1988) suggested.

### Measures

This study incorporated a self-report method to collect the data. Supervisory support was assessed using 16-items adopted from the perceived supervisory support scale (Kottke and Sharafinski, 1988). Examples of items are “My supervisor appreciates extra efforts from me.” The item responses were rated based on a six-point scale ranging from 1 = strongly disagree to 6 = strongly agree. Meanwhile, the reported reliability score for the scale was 0.84.

Self-efficacy was measured using six items from Rigotti et al. (2008). The response was rated on a scale (ranging from 1 = strongly disagree to 6 = strongly agree). One example of the item: “I feel prepared for most of the demands in my job.” The reported reliability score for the items was over 0.80.

Furthermore, OCB was measured using a 16-item scale from Lee and Allen (2002) on a six-point scale (1 = strongly disagree to 6 = strongly agree). For example, “I take action to protect the organization from the potential problem.” The reliability score of the OCB scale was 0.92.

### Data analysis

The study utilized partial least square (PLS) software by Ringle et al. (2015) to test the research hypotheses. Compared with covariance-analytical approach, the PLS-SEM was chosen due to the following reasons: (1) PLS-SEM is highly robust and reliable to provide high statistical power and is useful in resampling procedures for significant testing (Hair et al., 2017); (2) According to Astrachan et al. (2014), PLS-SEM can handle complex models with few endogenous and exogenous constructs and indicator variables, as well as non-normal data distributions; (3) PLS-SEM is effective with small sample sizes, whereas covariance-based SEM considers 200 to be the minimum sample size required for accurate model fit assessments (Hair et al., 2014); (4) PLS-SEM is the best test for theory development and testing (Astrachan et al., 2014) and it is well suited to the current study. To date, SmartPLS is the most popular variance-based structural modeling application in Management, Strategic Management, and Marketing due to its user-friendly interface and adaptable features (Astrachan et al., 2014; Ahmad et al., 2022). Thus, the use of PLS-SEM was justified.

The study also performed a two-stage procedure from Anderson and Gerbing (1988). First, the measurement model was analyzed to assess the relationship between the observed items and latent variables. Data were scrutinized through reliability (internal consistency and validity and convergent and discriminant validity). Secondly, structural model testing was conducted to specify the relationship between latent variables in the model and evaluated using the significance path coefficient and *R*^2^ measures.

## Results

### Descriptive results

Out of the 618 respondents, most public administrators were female (61.0%), while the remaining were male (39.0%). Most respondents were Malay (87.9%), followed by Chinese (6.1%), Indian (5.0%), and other races (1.0%). For marital status, most respondents were married (74.1%), followed by singles (22.5%), and 3.4% of the respondents were separated or divorced. The majority of respondents were bachelor’s degree graduates (64.7%), and only 35.3% of the respondents have a Master’s or PhD. Meanwhile, most respondents have worked for 6–9 years (34.6%), followed by over 10 years of working experience (33.2%), 20.9% of the respondents have 3–5 years of experience, while only 11.3% of the respondents work in 1–2 years tenure. Table 1 presents the respondents’ demographic profiles.

**TABLE 1:** Respondent demographic profile.

Demographic variables	Category	Frequency	Percentage
Gender	Male	241	39.0
	Female	377	61.0
Age	20–29 years	79	12.8
	30–39 years	363	58.7
	40–49 years	161	26.1
	Over 50 years	15	2.4
Race	Malay	543	87.9
	Chinese	38	6.1
	Indian	31	5.0
	Others	6	1.0
Marital status	Single	139	22.5
	Married	458	74.1
	Separated	6	1.0
	Divorced	15	2.4
Education	Bachelor’s degree	400	64.7
	Master’s degree	207	33.5
	PhD	11	1.8
Tenure	1–2 Years	70	11.3
	3–5 Years	129	20.9
	6–9 years	214	34.6
	Over 10 years	205	33.2

### Measurement model results

The first step involves evaluating the construct reliability and validity in the measurement model. The study assessed the factor loadings of each item, composite reliability (CR), and Cronbach’s alpha (see Table 2; Henseler et al., 2009). According to Hair et al. (2017), CR values above 0.70 is considered satisfactory and values below 0.60 indicate lack of consistency reliability. However, any items with factor loading between 0.4 and 0.7 will be excluded if the exclusion increases the CR of that particular variable (Hair et al., 2017).

**TABLE 2:** Internal consistency reliability and convergent validity.

Construct	Measurement item	Loading	CR	AVE
Self-efficacy	SE1	0.759	0.915	0.643
	SE2	0.818		
	SE3	0.787		
	SE4	0.806		
	SE5	0.812		
	SE6	0.825		
Organizational citizenship behavior	OCB10	0.713	0.914	0.516
	OCB11	0.729		
	OCB12	0.762		
	OCB13	0.765		
	OCB14	0.778		
	OCB15	0.757		
	OCB16	0.697		
	OCB1	Deleted		
	OCB2	Deleted		
	OCB4	0.618		
	OCB5	0.701		
	OCB7	0.650		
	OCB3	Deleted		
	OCB6	Deleted		
	OCB8	Deleted		
	OCB9	Deleted		
Supervisory support	Support1	0.679	0.947	0.559
	Support10	0.746		
	Support11	0.779		
	Support13	0.759		
	Support14	0.804		
	Support15	0.713		
	Support16	0.720		
	Support3	0.740		
	Support4	0.754		
	Support5	0.714		
	Support6	0.718		
	Support7	0.757		
	Support8	0.786		
	Support9	0.789		
	Support12	Deleted		
	Support2	Deleted		

CR, composite reliability; AVE, average variance extracted; OCB1, OCB2, OCB3, OCB6, OCB8, OCB9, Support12, and Support2 were deleted due to low loading.

All items loaded on each construct are relevant when the value exceeds the cut-off value of 0.7 (Hair et al., 2017), except supervisory support (Support2 and Support12) and OCB (OCB1, OCB2, OCB3, OCB6, OCB8, and OCB9) with loading less than 0.4. Therefore, all the constructs CR (see Table 2): supervisory support (0.947), self-efficacy (0.915), and OCB (0.914) in the study measurement model were satisfactory. Next, the convergent validity of the study was evaluated based on the item factor loading and average variance extracted (AVE). Henseler et al. (2009) stated that convergent validity is a set of indicators that represent one or similar underlying construct. Hair et al. (2017) recommended that the general rule of thumb for the AVE threshold value should exceed 0.50. Table 2 demonstrates that all constructs have an adequate AVE after removing all low loading items, whereas each construct can explain over 50% of its variance. Hence, convergent validity for the construct is verified.

The discriminant validity was assessed to evaluate the differences of each latent variable with its measurement variable or with other constructs (Hair et al., 2017). Two methods can be used to test discriminant validity: the Fornell-Larcker criterion and the heterotrait-monotrait ratio (HTMT) criterion (Henseler et al., 2015). According to Fornell-Larcker criterion, the AVE square root for all the variables should be greater than the others when compared diagonally (Hair et al., 2017). Table 3 presents that the AVE square root for self-efficacy (0.802) is higher than supervisory support with a 0.523 value. Therefore, the AVE value for OCB exceeds self-efficacy and supervisory support with an AVE value of 0.719. The HTMT criterion values below 0.85 (Hair et al., 2017) for all the constructs in Table 4 suggest that the study measurement model has satisfactory discriminant validity.

**TABLE 3:** Discriminant validity (Fornell and Larcker criterion).

Constructs	OCB	Self-efficacy	Supervisory support
OCB	**0.719**		
Self-efficacy	0.589	**0.802**	
Supervisory support	0.520	0.523	**0.748**

Bold figures represent high AVE square root values.

**TABLE 4:** Discriminant validity (HTMT Criterion).

Constructs	OCB	Self-efficacy	Supervisory support
OCB	–		
Self-efficacy	0.658	–	
Supervisory support	0.560	0.569	–

Criteria: Discriminant validity established at HTMT 0.85.

### Common method bias

Common method bias (CMB) has been employed in procedural and statistical approaches to ensure that CMB is not an issue in the study. In the procedural approach, the respondents’ anonymity and confidentiality were ensured. For instance, a pilot test was conducted upon the actual data collection and clear instruction was provided to facilitate the survey completion (Podsakoff et al., 2003). Moreover, the study employed Harman’s one-factor test to counter common method variance issues and to predict any potential threat to the study interpretations. All 38-items were tested using exploratory factor analysis in the SPSS Version 25. Resultantly, CMB was not an issue in the study, whereas the first factor makes up 40.77% of the variance, which did not count for most of the variance (Podsakoff et al., 2003).

### Structural model results

The structural model evaluates the relationship between latent variables and the measurement model together with the indicators (Chin and Saunders, 2009; Hair et al., 2017). Table 5 and Figure 1 showed that supervisory support is an important job resource and a significant contributor to employees’ OCB (β = 0.283, *t* = 6.358, *p* < 0.01). Thus, hypothesis H1 is accepted.

**TABLE 5:** The structural model results.

Hypothesis relationship	β	*t*-value	*p*-value	Decision	*R* ^2^
H1: Supervisory support–> OCB	0.283**	6.358	<0.01	Supported	0.415
Self-efficacy – > OCB	0.406**	9.334	<0.01	Supported	
H2: Supervisory support × Self-efficacy – > OCB	–0.069*	2.387	0.017	Supported	

*p < 0.05; **p < 0.01.

**FIGURE 2:**
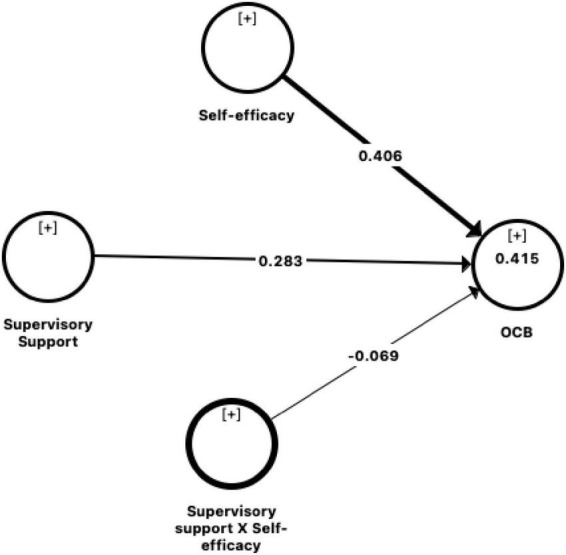
Structural model.

**FIGURE 3:**
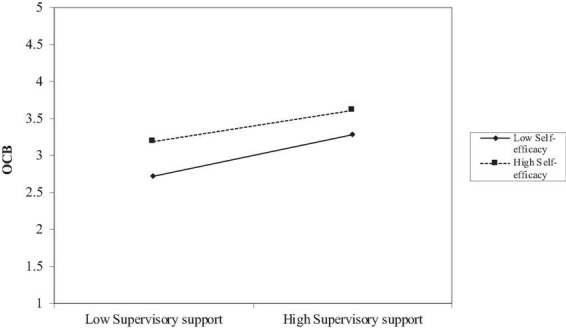
The moderating effect of self-efficacy on the relationship between supervisory support and OCB.

The H2 suggested the moderating role of self-efficacy between supervisory support and OCB. The following product indicator approach was utilized based on Chin et al. (2003). Specifically, the study predicted (1) the influence of supervisory support on OCB; (2) The direct impact of self-efficacy on OCB; and (3) the relationship between supervisory support and self-efficacy on OCB. The path analysis evaluation was done based on the value of t statistics (*t*-value) from path coefficient table (Table 5) with a threshold value is above 1.96 at a significant level *p*-value of 0.05 (*p* < 0.05) (Hair et al., 2017). Based on Figure 1, the results revealed that the relationship between supervisory support and OCB is moderated by self-efficacy (β = –0.069, *t* = 2.387, *p* < 0.05), such that the negative relationship is strongest when self-efficacy is highest. Table 5 depicts significant interaction effects within the model. Subsequently, the results were plotted to determine whether the interaction was consistent. The OCB was plotted at high (+ SD) and low (–SD) levels of supervisory support and self-efficacy as illustrated in Figure 2. A simple slope test was conducted to verify if the line slopes in the interaction plot were significantly different from 0. The analysis indicated that the relationship between supervisory support and OCB was significantly different for individuals who perceived high self-efficacy compared to low self-efficacy. Thus, H2 is accepted. The coefficient of determination of *R*^2^ represents an endogenous construct is explained by the coefficient variance from all exogenous constructs. According to Hair et al. (2017), *R*^2^-values above 0.75 are considered substantial, 0.50 considered as moderate and 0.25 considered a weak level of predictive accuracy. Table 5 indicates that supervisory support explained 41.5 per cent of the variance in OCB (*R*^2^ = 0.415). Based on Cohen’s (Cohen et al., 2000) calculation, the moderating effect size (*f*^2^) was only 0.01, which is considered small. Figure 3 depicts the relationship between supervisory support and OCB which vary significantly between high self-efficacy and low self-efficacy. The results demonstrate that although employees have low self-efficacy, their level of engagement in OCB activity increases steadily as supervisory support increases from low to high.

## Discussion

The study aims to examine the direct relationship between supervisory support and OCB and test the moderating effect of self-efficacy among Malaysian public sector employees. Resultantly, supervisory support indicated a significant positive relationship with OCB. The finding aligns with past research, such as (Hobfoll, 2002; Wang, 2014; Yadav and Rangnekar, 2015; Tremblay and Gibson, 2016; Abdullah and Marican, 2020), which highlighted the role of supervisory support in assisting employees in engaging in OCB. Summarily, employees who perceive supervisory support will be in a state of resource gain spiral (Hobfoll, 2002), which encourages them to invest in personal resources by engaging in extra-role behavior to gain further resources. Supervisory support causes employees to feel recognized while the instrumental, informative, and emotional support received would encourage employees to execute a higher level of performance and aid the organization for better productivity and effectiveness.

The findings revealed that employees with low and high self-efficacy would steadily increase engagement in extra-role activities, such as exhibiting OCB when they perceived higher supervisory support. Generally, employees have higher confidence and ability to effectively perform the tasks at hand (Somech and Drach-Zahavy, 2000) and engage in OCB activities despite low supervisory support. Similarly, a high self-efficacious individual is more engaged in OCB. Self-efficacy is a personal resource and self-motivating mechanism where competent employees are motivated to overcome a workplace obstacle (Guglielmi et al., 2012). Public administrators in governmental organizations are involved in a professional task and they must be firm with their decision-making, thus a supportive working environment is essential to boost their confidence. Public administrators manage various shareholders, such as superiors, co-workers, subordinates, and external parties. Therefore, belief in oneself is crucial to enhance their communication and decision-making skills to perform their tasks more proactively and aid the organization and colleagues without expecting reward or recognition.

### Theoretical implications

The findings of the current study extends the existing literature in the following ways. First, prior research has primarily focused on various sectors such as the private sector, academic institutions, and public services. Based on this gap, the current study findings extend current knowledge of OCB, particularly in the public sector setting. Moreover, this study contributes to the existing literature by investigating the role of the moderating variable in a non-western context, such as Malaysia. Malaysians have a high level of collectivism that is not rigid or constrained by rules, and issues can be resolved through good relationships with others (Suharnomo and Hashim, 2019). As a result, the current study suggests that employees with high self-belief may strengthen supportive supervisors in order to increase citizenship behavior. This supports the assertion that maintaining good relationships with others while having high self-confidence leads to employees volunteering to participate in activities that are not part of their job responsibilities such as OCB.

Second, the findings addressed the gap in existing OCB studies, which lack moderator elements and offer a new paradigm to investigate the organizational outcome. This study is a pioneer in elaborating the underlying mechanisms of self-efficacy as a moderating variable in the perception of supervisory support in relation to OCB. Using the dynamics of COR theory (Hobfoll et al., 2003; Cherian and Jacob, 2013; Halbesleben et al., 2014), the current study identified self-efficacy as an important buffering mechanism through which perceived support from supervisors can enhance employees’ citizenship behavior and this process is further strengthened when employees have high self-belief. Self-efficacy is considered one of the psychological capital (Cherian and Jacob, 2013; Halbesleben et al., 2014) and a personal resource (Hobfoll, 2001; Guglielmi et al., 2012) which can significantly contribute to work-related resources and behavioral outcomes. To the best of our knowledge, prior studies has not explored the role of self-efficacy as a moderator in the relationship supervisory support and the OCB relationship.

Third, the results suggested that supervisory support is significantly positive with OCB, thus confirming past empirical findings (Yadav and Rangnekar, 2015; Tang and Tsaur, 2016; Abdullah and Marican, 2020). Supervisory support plays a significant role in influencing employees’ extra-role behavior. Supervisory support can motivate employees to perform beyond their current responsibilities by providing an active assessment and continuous feedback to ensure organizational effectiveness. Hence, supervisors promoting “power to authority equity” is crucial to improve power-sharing with their subordinates. Therefore, supervisors can increase support for the employees, make them feel appreciated, view the organization as a supportive entity, and be willing to participate and engage in extra-role behavior, such as OCB.

Fourth, this work extends the application of a rigorous methodological approach to management research. Although moderation research is prevalent in management studies, prior scholars are increasingly proposing such rigorous methods to better comprehend today’s management difficulties and complex reality (Hair et al., 2017). The research model suggests the possibility of employing the structural equation modeling method in a systematic manner, which has been demonstrated to be an efficient analysis tool. This is accomplished by modeling the association between supervisory support and OCB using self-efficacy and supervisory support as individual level variables and OCB as an organizational level variable. Therefore, this study provides methodological implications to consider moderation relationship between supervisory support and OCB.

### Managerial implications

The finding that a supportive supervisor improves employees’ OCB and is reinforced by individuals’ self-belief suggests that creating a supportive work environment is critical for public organizations. Managers should use training interventions to encourage supervisors to recognize and value employees’ contributions to organizational improvement. Supervisors should be trained to support and treat their subordinates fairly in order to foster trust in their managers. Furthermore, an organization can foster a supportive culture by cultivating a healthy and positive supervisor-employee relationship. This will encourage job involvement and participation, which will not only result in a positive outcome but will also aid in the prevention of a variety of problems such as turnover intentions, low self-confidence, absenteeism, job dissatisfaction, and poor performance. Hence, public sector organizations may benefit from interventions that promote OCB among public sector employees. As a result, the current study has important implications for identifying the presence of supervisory support and the resulting improvement in OCB, as well as fostering employee self-efficacy.

The finding that self-efficacy buffers the significant impact of supervisory support on OCB suggests that self-efficacy can at least strengthen the low level of supportive supervisors. This highlights the importance of increasing employees’ self-efficacy as a means of reducing counter-productive behavior and encouraging OCB among employees. Public sector organizations may want to consider development programs like training, consulting, and coaching, as well as behavioral modeling and soft skills training. Such development initiatives will effectively encourage and assist employees in increasing self-confidence, resulting in availability and willingness to contribute to citizenship behavior.

### Limitations and recommendations

There are several limitations to the current study. First, current findings should not be generalized to other contexts. The research was carried out in selected public sector organizations within the Malaysian federal government. Several differences may exist in terms of work environment, job scope, and individual characteristics, which are reinforced by the culture of each organization. Hence, participants in each organization differ in cultural and social contexts, and their interpretations of the constructs differ from those in other organizations and countries. As a result, the findings should be restricted to public-sector organizations. Future research could focus on other public sector organizations, such as state government, local governments, or city council levels, as well as different industries, such as the private sector, academic institutions or non-government organizations, for a more in-depth investigation, allowing for a better generalization of these findings to other contexts.

Second, self-efficacy is the only moderator used in the relationship between supervisory support and OCB. This does not imply that this is the only moderating mechanism underlying the supervisory support OCB relationship. Future research should look into the impact of other moderators on this process. In addition, future research may investigate the differential impact of supervisory support and self-efficacy on the various dimensions of OCB such as altruism, conscientiousness, civic virtue, courtesy, and sportsmanship. The findings of this study could serve as the baseline for future studies that investigate the effects of OCB on the population as a whole.

Furthermore, the current study used a cross-sectional survey that was conducted over a single time period. This precludes any conclusions about the causality implied by the relationship. A longitudinal research design could establish definitive conclusions about cause-and-effect made at different points in time (Green et al., 2016). Although CMB was not a major concern in the study, future research should consider using multi-source data collection methods and a mixed method research design. For example, data can be collected from supervisors, coworkers, colleagues, and subordinates (Podsakoff et al., 2012) and they can be interviewed to determine the exact nature of employees’ self-efficacy and supervisory support in OCB. Furthermore, data from other parties, such as supervisors or coworkers, would allow for a more complete understanding of the supervisory support and individual self-efficacy implications for OCB in the public sector. Nonetheless, the findings of this study will be useful to practitioners who want to encourage a supportive environment and improve employees’ self-efficacy and OCB.

## Conclusion

According to the findings, self-efficacy moderates the relationship between supervisory support and OCB. The findings suggest that self-efficacy plays a significant role in the encouragement of supportive supervisors and workplace behavior such as OCB. The current study adds to the literature on organizational behavior, specifically employee performance, by incorporating the moderating effect of self-efficacy. The study fills a gap in the existing literature, particularly in the context of OCB, and thus adds to our understanding of the impact of various types of resources, such as self-efficacy and supervisory support, on citizenship behavior in Malaysian government organizations. As a result, the goal of this research is to encourage government organizations to develop and improve their current policies in order to successfully provide a better work environment and, as a result, increase employee productivity and overall performance.

## Data availability statement

The raw data supporting the conclusions of this article will be made available by the authors, without undue reservation.

## Ethics statement

Ethical approval was not provided for this study on human participants because the article’s survey did not identify specific individuals, and participation was completely voluntary. Therefore, ethical review and approval was not required in accordance with the local legislation and institutional requirements. The patients/participants provided their written informed consent to participate in this study.

## Author contributions

MA: conceptualization, formal analysis, investigation, methodology, project administration, and writing. WW: writing—review and editing. Both authors contributed to the article and approved the submitted version.
